# The Levantine jellyfish *Rhopilema nomadica* and *Rhizostoma pulmo* swim faster against the flow than with the flow

**DOI:** 10.1038/s41598-019-56311-3

**Published:** 2019-12-30

**Authors:** Dror Malul, Tamar Lotan, Yizhaq Makovsky, Roi Holzman, Uri Shavit

**Affiliations:** 1 Civil and Environmental Engineering, Technion IIT, Haifa, 32000 Israel; 2 Department of Marine Biology, The Leon H. Charney School of Marine Sciences, University of Haifa, 3498838 Haifa, Israel; 3 The Department of Marine Technologies, The Leon H. Charney School of Marine Sciences, University of Haifa, 3498838 Haifa, Israel; 4 School of Zoology, Faculty of Life Sciences, Tel Aviv University, Tel Aviv, 69978 Israel; 5 The Inter-University Institute for Marine Sciences, POB 469, Eilat, 88103 Israel

**Keywords:** Motility, Fluid dynamics

## Abstract

Jellyfish locomotion and orientation have been studied in the past both in the laboratory, testing mostly small jellyfish, and in the field, where it was impossible to control the seawater currents. Utilizing an outdoor water flume, we tested the locomotion of jellyfish when swimming against and with currents of up to 4.5 cm s^−1^. We used adult jellyfish from two of the most abundant species in the eastern Mediterranean, *Rhopilema nomadica* and *Rhizostoma pulmo*, and measured their pulsation frequency and swimming speed relative to the water. While pulsation frequency was not affected by the water velocity, jellyfish swam faster against the current than with it. This finding suggests that jellyfish possess a sensory ability, whose mechanism is currently unknown, enabling them to gauge the flow and react to it, possibly in order to reduce the risk of stranding.

## Introduction

Although jellyfish are often regarded as planktonic drifters, many studies have shown that they possess a surprising range of swimming capabilities. Kinematic studies have revealed how oblate jellyfish swim by paddling their bell and shedding vortex rings^1–3^, and how prolate jellyfish swim by generating spewing jets^4,5^. Jellyfish modes of propulsion have been shown to be energetically efficient compared to all other recorded metazoans^2^. Numerical investigations have also suggested that jellyfish pulsate in their natural resonance frequency, consequently achieving higher velocities^6^. These and other locomotion skills have inspired the design of biomimetic swimming robots^7–9^. Some studies have demonstrated that jellyfish modulate their swimming as a reaction to their environment. They were shown to search up and down the water column for food^10,11^, pulsate asymmetrically to counteract shear flows^12^, and change their orientation in reaction to tide regimes, possibly to reduce the risk of stranding^13^.

The ability of jellyfish to actively change their swimming speed and direction in response to ambient conditions was suggested to be crucial for their chances of survival and bloom formation^13^. However, sensing the ambient water velocity requires special abilities. Sensing shear away from the seafloor or water surface, for example, is inefficient, as the speed of the jellyfish relative to the current remains constant both when the jellyfish drifts passively with the flow and when it moves at a constant speed relative to the flow, regardless of the velocity and direction of the current. Therefore, the hypothesis of the present study was that under steady and uniform conditions, jellyfish will be unable to sense the absolute velocity of the surrounding flow (i.e., the water velocity with respect to the ground frame of reference). Nonetheless, some studies suggest that jellyfish may have the ability to indirectly assess and react to the ambient velocity (or to its own absolute swimming speed, i.e., relative to the ground), using cues such as the magnetic field, infrasound signals, shear when swimming near the bottom or the surface, and wave orbital motion^13,14^.

Many of the relevant studies focused on relatively small jellyfish, mostly *Aurelia aurita*, and were performed under lab conditions with no flow^1,2^. Other studies investigated the motion of jellyfish under field conditions. Shanks and Graham^14^ examined the swimming of jellyfish across a submerged breakwater and determined the jellyfish velocity relative to the ground by measuring its displacement during ∼1 *min*. However, they neglected the current velocity and did not provide the swimming speed relative to the water velocity. Fosstte *et al*.^13^ attached accelerometers to live jellyfish and measured their vertical swimming speed. They used the displacement of buoys as a proxy for the water speed and measured the direction of jellyfish swimming relative to the direction of the flow. However, they did not report on the jellyfish swimming speed relative to that of the water and did not test the jellyfish relative speed when swimming with or against the flow.

**Figure 1:**
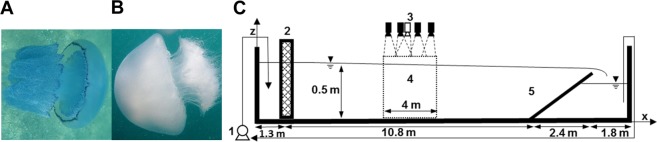
The study animals and a side view sketch of the experimental apparatus. (**A**) *Rhizostoma pulmo* (**B**) *Rhopilema nomadica*. (**C**) 1. Pumps 2. Flow straightener 3. Video camera array: black – Sony cameras, white – Canon camera 4. Experimental area 5. Hydraulic weir. The scheme aspect ratio is not to scale.

Here, we studied two of the most prominent local species of the eastern Mediterranean Sea, Rhizostoma pulmo (Fig. 1A) and Rhopilema nomadica^15^ (Fig. 1B), and recorded their swimming speed and pulsation frequency both when swimming with the flow and when swimming against it, under various ambient flow velocities. Using an onshore, outdoor water flume, we were able to create a unidirectional flow while eliminating most of the other flow components present in the field such as tides and surface waves. Other physical gradients, like density and temperature gradients, were also avoided in our experiments, which focused on the magnitude and direction of the ambient velocity as the only experimental variable. We selected two jellyfish species that belong to the same family and order, feature the same prolate design, and swim by symmetrically paddling their bell. The species differ in distribution: *R. pulmo* is found throughout the Eastern Atlantic Ocean, Mediterranean Sea and the Black Sea, while *R. nomadica* is found mainly in the Eastern Mediterranean^16^, where it has been flourishing for the last few decades in frequent seasonal swarms that constitute a significant nuisance for infrastructure, tourism, and aquaculture, and bear dire economic implications^17–20^. *Rhizostoma octopus*, a North Sea species that was studied by Fossette *et al*.^13^, is closely related and morphologically similar to *R. pulmo*.

## Materials and Methods

### Study organisms

Adult medusa-stage *R. pulmo* and *R. nomadica* specimens (bell diameters: *R. pulmo* 10–20 *cm*; *R.nomadica* 10–14 *cm*; Fig. 1A,B) were collected between July and September 2015 from the Haifa bay area in a series of five field collections. Specimens were collected at an approximate distance of 1 *km* from the shore and were tested in the experiments on the same day of collection. The jellyfish were collected individually by a swimmer using a 12-liter round bucket to avoid damage to the animals and then gently transferred to a 60–80 liter container on the boat. Upon arrival to shore, jellyfish were left to acclimate for half an hour in an outdoor seawater flume, 18 *m* long, 2 *m* wide and 1.2 *m* deep, with constant replenishment of seawater, while monitored carefully by visualization. Only actively swimming jellyfish that did not show any external signs of injury were transferred to the experimental apparatus. Bell diameters were measured from the footage at maximal aperture of the bell during the pulsation period.

### Experimental water flume

A second 18 *m* long, 2 *m* wide and 1.2 *m* deep outdoor water flume, equipped with two circulating water pumps, was refitted to create a uniform turbulent flow field (Fig. 1C). The flume cross-section was narrowed to 50 cm and shallowed to 50 cm. A honeycomb-like flow straightener was installed at the inlet and a hydraulic weir was placed near the flume exit. The central section of the flume (4 m long) was designated as the experimental area, away from the flow straightener and weir, to minimize their effect on the flow field in and to allow the jellyfish to swim a substantial distance freely before arriving at the experimental area. To continuously replenish the water in the flume, we added an external seawater inlet and a drain positioned near the outlet in order to maintain a constant water depth in the outlet container. The constant recirculation with seawater guaranteed that the flume water temperature was kept similar to that in the sea, namely 30–31 °C in July and August and 28 °C in September.

The flow rate, *Q* [*L*^3^*T*^−1^], of each pump and the external inlet were recorded separately, and the average water velocity in the experimental area was calculated as *U*_*flume*_ = *Q*/*A* where *A* is the cross-sectional area. The external water inlet produced an average velocity of 0.5 *cm s*^−1^ and each pump increased the velocity by 2 *cm s*^−1^. Combining the three modes of pump operation (one pump, two pumps, and no pumps) and the two possible directions of jellyfish swimming (with the ambient current and against it) we could record segments of swimming under six different swimming conditions: swimming in the direction of an ambient current of 0.5, 2.5, and 4.5 *cm s*^−1^ and swimming against a current of 0.5, 2.5, and 4.5 *cm s*^−1^.

### Videography and calibration

The experimental area was filmed from above using four stationary cameras (Sony ICX693 CCD @ 25 FPS) positioned above the flume center line with a slight overlap. A fifth camera (Canon G12, @ 24FPS) was positioned near one of the other cameras and was used to record voice notes during the experiments. Prior to each experiment, a metal grid (5 *cm* × 15 *cm* spacing) was submerged in the water in front of the camera array at three depths (top: z = 50 *cm*, middle: z = 25 *cm*, and bottom: z = 0 *cm*) and used to calibrate the coordinate system of the experimental area. The pixel location of each grid intersection was recorded, and a linear interpolation was used to convert every pixel location to a matching location of the grid intersections (cm) for the three depths.

### Tracking the jellyfish location

*R. pulmo* is a blue-hued jellyfish (Fig. 1A) and *R. nomadica* is white and more transparent (Fig. 1B), making it more difficult to see in the video. Due to *R. pulmo* distinct color, we could utilize an automatic algorithm when tracking it (as described in the Supplementary Information, Fig. S1). The location of *R. pulmo* was chosen as the center of a polygon (Eq. S1) created by an outline tracing procedure. Movie S1 shows the outline tracking results as acquired by the automatic procedure for one of the *R. pulmo* individuals. The bell tip of *R. nomadica* was manually tracked frame-by-frame.

Jellyfish were introduced into both ends of the flume and were allowed to swim freely up and down the flume. For data analysis, only segments of “free swimming”, longer than three seconds, during which the jellyfish did not hit the walls of the flume or changed elevation in the water column were considered and analyzed as discrete swimming segments (Fig. S2). Examples of two swimming segments are shown in Fig. S2A. A total of 54 swimming segments of *R. nomadica* and 159 of *R. pulmo* were tracked. Table S1 presents the distribution of these segments across jellyfish size and water velocity in the flume.

### Calculation of jellyfish swimming speed

The location plots for each swimming segment were smoothed twice using a moving average filter (with a span of 16 frames). The displacement of the jellyfish (Δ*s* [*L*]) between two consecutive frames, relative to the water flow and along the jellyfish axis of motion, was calculated as follows,

1 $$\Delta s=\alpha \sqrt{{(\Delta X-\Delta t\cdot {U}_{flume})}^{2}+\Delta {Y}^{2}}$$

where (*X*, *Y*) is the jellyfish location in the (*x*, *y*) horizontal plane of the coordinate system and Δ*X* [L] and Δ*Y* [L] describe the displacement between two consecutive frames in the *x* and *y* directions, respectively. Both (*X*, *Y*) and (*x*, *y*) are defined relative to the ground frame of reference (i.e. the camera reference). The product of the time interval between two frames, Δ*t* [*T*], and the velocity of the water in the flume, *U*_*flume*_, is used to transform the displacement defined by the camera frame of reference to the displacement relative to the moving water frame of reference.

When a jellyfish swam just below the water surface or near the bottom of the flume, its center of mass did not lie on the surface or at the bottom where calibration images were taken. To correct for this potential discrepancy we multiplied the displacement by a depth correction factor *α*, which is the ratio between the cm-to-pixel ratio at the top or bottom and the cm-to-pixel ratio half a jellyfish diameter away from the top or bottom, which was obtained via linear interpolation (*α* ranged between ∼0.92 for the bottom and ∼1.08 for the top). Jellyfish that swam in the middle depth of the flume did not need a correction (*α* = 1). Displacement, relative to the water frame of reference, between two frames was summed along the segment of swimming to obtain the distance along the jellyfish’s axis of motion relative to the water, *s* [*L*]. The jellyfish average swimming speed in the segment (${\overline{U}}_{jelly}\,[L\,{T}^{-1}]$) was taken as the slope of a linear fit of *s*(*t*), where *t* is time (two examples are shown in Fig. S2B,C).

### Calculation of pulsation frequency

Calculation of the pulsation frequency of *R. pulmo* was obtained by a Fast Fourier Transform (FFT). As the jellyfish pulsates, it contracts and relaxes, altering its projected surface area. The time history of the projected surface area (Fig. S3A and Movie S1) was analyzed using FFT and the most dominant frequency was taken as the frequency of pulsation for each swimming segment (*f*_*jelly*_[*T*^−1^], Fig. S3B). The frequency of *R. nomadica* was obtained manually by timing every swimming segment and counting the number of full periods it completed.

### Statistical analysis

Values for the different segments of swimming were weighted averaged (Eqn. S2–S5) according to flume velocity, where the weighting parameter was the duration of the segment (*t*_*k*_ [*T*]) relative to the sum of durations of all the segments that contributed to the average (Table S1). We used multiple regression to test the effect of water velocity in the flume and jellyfish bell diameter (independent variables) on the swimming speed of the jellyfish (dependent variable). Both independent variables were treated as continuous predictors. A separate analysis was carried out for the two species. A similar analysis was conducted to determine the effect of the water velocity in the flume and the jellyfish size on the pulsation frequency of the jellyfish (dependent variable). The assumptions of linear regression (namely normal distribution of the residuals) were verified for each model.

## Results and Discussion

Jellyfish were allowed to swim freely in the experimental flume with and against the flow while examining their behavior under three different mean velocities: ±0.5 *cm s*^−1^, ±2.5 *cm s*^−1^ and ±4.5 *cm s*^−1^ (+ with the flow and - against the flow). The jellyfish trajectories were recorded along discrete swimming segments and their swimming speed (relative to the water) and pulsation frequency were calculated.

Averaging all swimming segments, *R. pulmo* swimming speed relative to the water was measured as 8.69 *cm s*^−1^ (*n* = 159, *SD* = 1.56 *cm s*^−1^), with an average pulsation frequency of 1.22 *Hz* (*n* = 153, *SD* = 0.15 *Hz*). In comparison, the swimming speed of *R. nomadica* was slower and equal to 6.70 *cm s*^−1^ (*n* = 54, *SD* = 1.87*cm s*^−1^), pulsating at almost the same frequency as *R. pulmo*, and measured as 1.23 *Hz* (*n* = 54, *SD* = 0.11 *Hz*).

**Figure 2:**
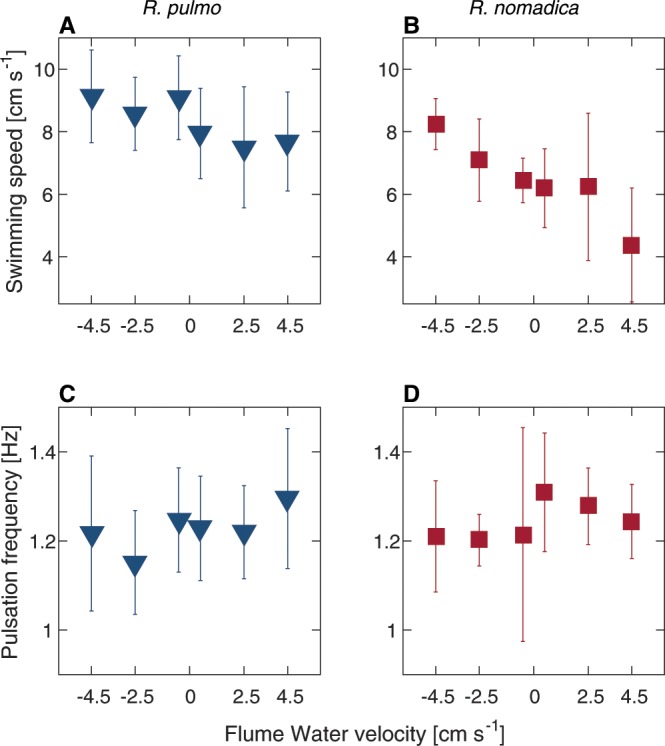
Swimming speed and pulsation frequency of *R. pulmo* (blue) and *R. nomadica* (red) under varying water velocity conditions. (**A**,**C**) Swimming speed and pulsation frequency (respectively) of *R. pulmo* in six water velocity conditions. (**B**,**D**) Swimming speed and pulsation frequency (respectively) of *R. nomadica* in six water velocity conditions. (A–**D**) Error bars display ± one standard deviation.

**Figure 3:**
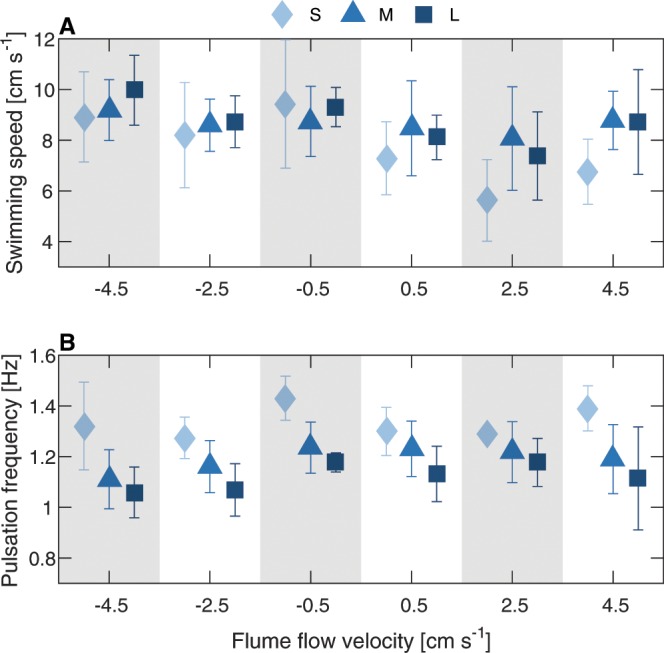
Swimming speed and pulsation frequency of *R. pulmo* under varying water velocity, sorted by size group. Swimming speed (**A**) and pulsation frequency (**B**) under six water velocity conditions. Error bars display ±  standard deviation. The results were binned and weighted averaged according to jellyfish bell diameter, defined as small) (S) 10−13 *cm*, medium)M) 14−16 *cm*, and large (L) 17−20 *cm*.

For both species, water velocity in the flume had a statistically significant effect on the jellyfish swimming speed (multiple regression: *P* < 0.001 for both species; Fig. 2A,B; Table S2). In general, both species achieved higher speeds when swimming against the flow than when swimming with it. The effect was more pronounced for *R. nomadica*, which swam ~25% faster against the flow (8.25 *cm s*^−1^ at −4.5 *cm s*^−1^) and ~35% slower with the flow (4.4 *cms*^−1^ at +4.5 *cm s*^−1^), compared with the overall average of 6.7 *cm s*^−1^. *R. pulmo* displayed a more subtle response to the flow direction. It swam only ~5% faster against the flow (9.12 *cm s*^−1^ at −4.5 *cm s*^−1^) and ~11% slower with the flow (7.7*cm s*^−1^ at +4.5*cm s*^−1^) compared with the overall average of 8.69 *cm s*^−1^. Bell diameter had a significant effect on the jellyfish swimming speed only for *R. Pulmo* (multiple regression: *P* < 0.007), with smaller jellyfish swimming more slowly (Fig. 3). Bell diameter did not have a significant effect on the swimming speed of *R. nomadica* (multiple regression: *P* > 0.2); however, the size range in *R. Pulmo* was much broader than for *R. nomadica*.

For both species, there was no significant effect of water velocity on jellyfish pulsation frequency (multiple regression: *P* > 0.09 for both species; Fig. 2C,D; Table S2). Average pulsation frequencies at the four flume velocities deviated from the overall mean pulsation frequency by less than 7%. However, size had a significant effect on pulsation frequency for both species (multiple regression: *P* < 0.014 for *R. nomadica* and *P* < 0.001 for *R. pulmo*). Generally, pulsation rate decreased with size (Fig. 3B), reflecting a trend that is in agreement with previous studies^21^.

Our results contradicted our original hypothesis that under uniform unidirectional flow jellyfish would swim at a relative velocity that is independent of the flow direction and velocity. This hypothesis was based on the current knowledge of jellyfish sensory abilities and hydrodynamic considerations. Previous studies have shown that jellyfish probably have the ability to sense gravity, hydrostatic pressure^22,23^, and light^22,24,25^, which may allow them to orient up and down the water column. Jellyfish were also suggested to be able to sense changes in the flow around them: they were observed to stay below turbulent regions, possibly signaled by pressure and velocity fluctuations generated by the turbulent flow^26^. They were also observed to align their swimming direction with the direction of waves^14^. Although the sensory mechanism underlying these behaviors is unclear, such behaviors have been widely observed and generally accepted to be a response to sensory stimuli^26^. The common feature of these sensing behaviors is that of the ability to identify variations in time (e.g., turbulence) or in space (e.g., shear flow). The flow profile created by waves, for example, is characterized by a time-dependent oscillating water velocity profile that potentially serves as a mechanical stimulus sensed by the jellyfish. It is unlikely, however, that jellyfish possess the ability to sense the direction and magnitude of the ambient velocity when it is steady and uniform. When drifting with the current, an external cue is needed in order to determine the direction of the flow relative to the ground. The jellyfish is exposed to the relative velocity of the water, which is a combination of the water velocity and the jellyfish swimming speed, both relative to the ground. Consider the same jellyfish experiencing the following scenarios: in one, it is swimming at 4 *cm s*^−1^ (relative to the ground) against a uniform 4 *cm s*^−1^ flow, and in the other it is swimming at 12 *cm s*^−1^ (relative to the ground) but in the direction of a unidirectional flow of 4 *cm s*^−1^. In both cases the jellyfish experiences a relative velocity of 8 *cm s*^−1^ and will not be able to distinguish between the two. However, as our results show, jellyfish swim faster when against the flow, suggesting that they can sense the difference between the two. These findings suggest that jellyfish employ a sensory mechanism, currently unknown, to sense the direction and intensity of the flow.

Fossette *et al*.^13^ suggested that jellyfish in the field may be able to assess flow direction using wave motion, water-air interface shear, or magnetic cues. None of which are good sensory candidates in our experimental setup. First, our onshore flume generated no waves; second, the water surface in our apparatus was shielded by the walls of the flume (see Movie S1), which virtually eliminated air flow velocities at the water surface; and third, magnetic cues, if indeed these are accessible to the jellyfish, would allow them to sense an horizontal displacement of several kilometers at best. Our experimental results show that jellyfish might be able to sense the direction of the absolute flow (relative to the ground frame of reference), regardless of these previously suggested mechanistic paths. However, given the size of some of the jellyfish we used, relative to the flume width, these species might have sensed the shear generated by the velocity profiles in the flume itself.

Increasing the swimming speed with respect to the moving water frame of reference improves processes such as mass transfer, encounter rates with prey and escape strategies. However, these potential improvements are insufficient to justify a swimming preference whether swimming with or against the flow. Therefore, regardless of the sensory mechanism, our results appear to reinforce the suggestion that jellyfish swim countercurrent as a means of avoiding stranding. The findings that jellyfish swim faster against the current, together with previous observations of countercurrent swimming preferences, suggest that this behavior is advantageous in terms of individual fitness.

## Supplementary information

Supplementary Information 

Supplementary Movie S1.
