# Supplementary material for: The Levantine jellyfish Rhopilema nomadica and Rhizostoma pulmo swim faster against the flow than with the flow
Source: Sci Rep. 2019 Dec 30;9:20337. doi: 10.1038/s41598-019-56311-3 (PMC6937341; doi:10.1038/s41598-019-56311-3)
Supplement: Supplementary file 1 — Supplementary Information [file 41598_2019_56311_MOESM1_ESM.docx]

**Supplementary information for**

**The Levantine jellyfish *Rhopilema nomadica* and *Rhizostoma pulmo* swim faster against the flow than with the flow**

Dror Malul^1,5,*^, Tamar Lotan^2,*^, Yizhaq Makovsky^2,3^, Roi Holzman^4,5^ and Uri Shavit^1^

^1^ Civil and Environmental Engineering, Technion IIT, Haifa 32000, Israel.
^2^ Department of Marine Biology, The Leon H. Charney School of Marine Sciences, University of Haifa, 3498838 Haifa, Israel.

^3^ The Department of Marine Technologies, The Leon H. Charney School of Marine Sciences, University of Haifa, 3498838 Haifa, Israel
^4^ School of Zoology, Faculty of Life Sciences, Tel Aviv University, Tel Aviv 69978, Israel.
^5^ The Inter-University Institute for Marine Sciences, POB 469, Eilat 88103, Israel.

^*^ Corresponding authors: Dror Malul <malolds@technion.ac.il>, Tamar Lotan <lotant@univ.haifa.ac.il>

This supplementary information elaborates on the technical aspects of tracking the jellyfish and on calculating the velocities and frequencies. Videos of jellyfish swimming upstream and downstream along experimental apparatus were manually cut into shorter segments of swimming where the jellyfish did not change elevation in the water column and did not hit the walls of the flume. The location of the jellyfish was then tracked in these shorter segments. Table S1 lists the number of segments by species, flow condition and size. Jellyfish of the species *R. nomadica* were tracked manually and jellyfish of the species *R. pulmo* were tracked automatically using the fact that this species has a blue color that could be distinguished from the background using simple image processing tools. Image processing allowed us to obtain the outline of the jellyfish and from it calculate jellyfish swimming speed and pulsation frequency. *R. nomadica* was too transparent to be easily separated from the background so it was tracked manually. [Movie S1](https://www.youtube.com/watch?v=IT671fcX6to) demonstrates the result of jellyfish outline tracking (green outline), jellyfish location (green dot), cumulative displacement with time and the projected area of the jellyfish which is representative of the pulsation period.

| **Table S1. Summary of the number of digitized segments for each size group of jellyfish and flume flow velocity.** Number of segments analyzed is different for frequency and speed for *R. pulmo* because in some segments the frequency could not be determined accurately from the video. *R. pulmo* swimming segments were binned by jellyfish bell diameter, defined as small )S($10-13 cm$, medium )M) $14-16 cm$ and large (L) $17-20 cm$. Realizations of the *R. nomadica* are not divided to size groups due to the relative homogeneity of their diameter.   \|  \| Flow velocity $[cm s^{-1}]$ \| $-4.5$ \| \| \| $-2.5$ \| \| \| $-0.5$ \| \| \| $0.5$ \| \| \| $2.5$ \| \| \| $4.5$ \| \| \| \| --- \| --- \| --- \| --- \| --- \| --- \| --- \| --- \| --- \| --- \| --- \| --- \| --- \| --- \| --- \| --- \| --- \| --- \| --- \| --- \| \| ***R. nomadica*** \| Frequency & velocity \| 10 \| \| \| 15 \| \| \| 6 \| \| \| 3 \| \| \| 10 \| \| \| 10 \| \| \| \| ***R.  pulmo*** \|  \| S \| M \| L \| S \| M \| L \| S \| M \| L \| S \| M \| L \| S \| M \| L \| S \| M \| L \| \| Frequency \| 31 \| 32 \| 8 \| 4 \| 13 \| 7 \| 3 \| 7 \| 7 \| 5 \| 4 \| 4 \| 2 \| 7 \| 3 \| 8 \| 6 \| 2 \| \| Velocity \| 32 \| 33 \| 8 \| 4 \| 13 \| 7 \| 4 \| 8 \| 7 \| 5 \| 4 \| 4 \| 2 \| 8 \| 3 \| 8 \| 7 \| 2 \| |
| --- | --- | --- | --- | --- | --- | --- | --- | --- | --- | --- | --- | --- | --- | --- | --- | --- | --- | --- | --- | --- | --- | --- | --- | --- | --- | --- | --- | --- | --- | --- | --- | --- | --- | --- | --- | --- | --- | --- | --- | --- | --- | --- | --- | --- | --- | --- | --- | --- | --- | --- | --- | --- | --- | --- | --- | --- | --- | --- | --- | --- | --- | --- | --- | --- | --- | --- | --- | --- | --- | --- | --- | --- | --- | --- | --- | --- | --- | --- | --- | --- | --- | --- | --- | --- | --- | --- | --- | --- | --- | --- | --- | --- | --- | --- | --- | --- | --- | --- |

*Image processing*

Images were converted from the standard RGB color space (Fig. S1A) to HSV^1^ in order to manually choose a blue hue value to distinguish the jellyfish from the surrounding background (Figs. S1B and S1C). Hue values selection was done manually for each video because the lighting conditions varied notably from video to video and between experiment days. Image noise was removed (Fig. S1D) by morphological closing^2^ (4 pixel radius disk) and a 2D median filters ^3^ $10\times10$ pixels square neighborhood). Finally, edge detection (Fig. S1E) was applied by using the Sobel method^4^ and the outline of the jellyfish was marked using the Moore neighbor tracing algorithm^5^ (Jacob stopping criteria). Jellyfish location ($\boldsymbol{X}_{\boldsymbol{jelly}}=(X_{jelly},Y_{jelly})$) was chosen as the center of the polygon created by the outline according to equation S1:

| $\boldsymbol{X}_{\boldsymbol{jelly}}=\sum_{i=1}^{N} \boldsymbol{X}_{\boldsymbol{i}}/N$ | (S1) |
| --- | --- |

where $N$ is the number of points defining the outline and $\boldsymbol{X}_{\boldsymbol{i}}$ $=(X_{i},Y_{i})$ is the location of the $i$th point along the outline (60 points gave adequate resolution).


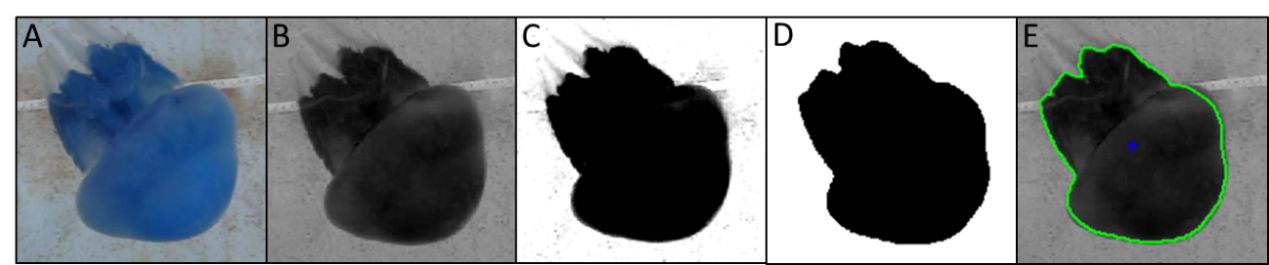


*

**Fig. S1. Image processing procedure.** (A) An example of a raw image. (B) The image hue layer shown in HSV color space. (C) A binary representation generated by manually selecting a hue threshold value. (D) The image after applying the morphological closing and the 2D median filters. (E) The jellyfish edge (green) and the center of the edge polygon (red asterisk, representing the jellyfish location) superimposed on the image shown in B.

*Jellyfish swimming speed and pulsation frequency calculation*

Jellyfish were tracked in the camera frame of reference (Fig. S2A). Water flow was taken into account (using Eq. 1) to convert the location to cumulative displacement in the water frame of reference (Fig. S2 A and B). Figure S2A shows jellyfish trajectories as captured in the camera frame of reference for the two jellyfish species: *R. pulmo* (blue triangles) covers a shorter distance on the camera frame than *R. nomadica* (red squares), however because *R. pulmo* swims against the current it actually achieves higher speed in the moving water frame of reference ($6.4 cm s^{-1}$ compared with $5.4 cm s^{-1}$).

**
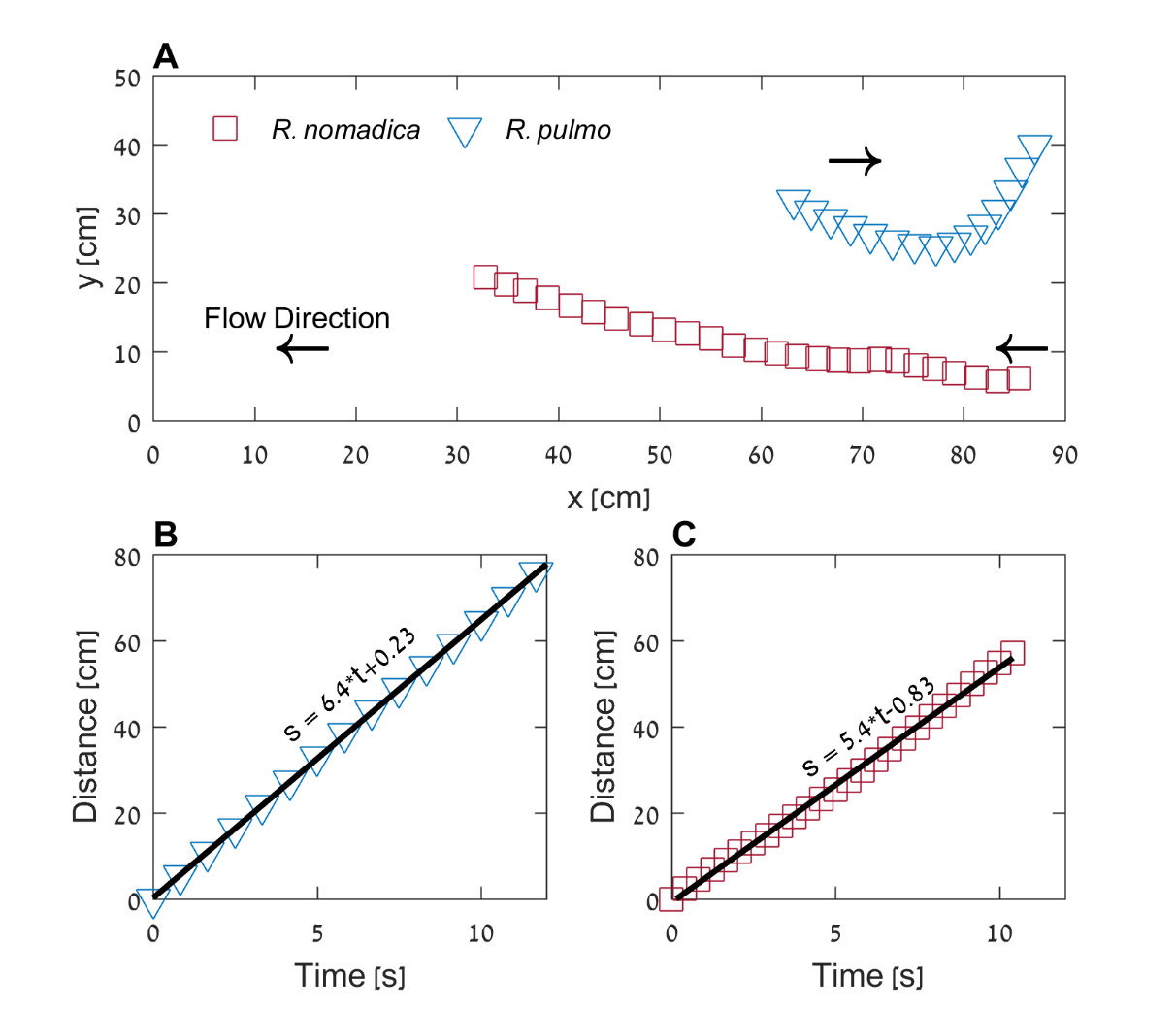
**

**Fig. S2. Obtaining jellyfish segment velocity.** (A) Location plot for two swimming segments in the ground frame of reference: *R. pulmo* (Blue triangles; every tenth location is shown), *R. nomadica* (Red squares; every twentieth location is shown). The arrows indicate the direction of motion of the jellyfish and the flow. (B-C) Cumulative displacement curves, in the water frame of reference, for the swimming segments shown in A: B - *R. pulmo*, C - *R. nomadica*. Black line – linear fit (B: $r^{2}=0.99$, C: $r^{2}=0.99$).

The time plot of the projected surface area of the polygon was used to calculate the jellyfish pulsation period (Fig. S3A). FFT was used to obtain the power-frequency as demonstrated in figure S3B, and the dominant frequency was chosen as the jellyfish pulsation frequency.


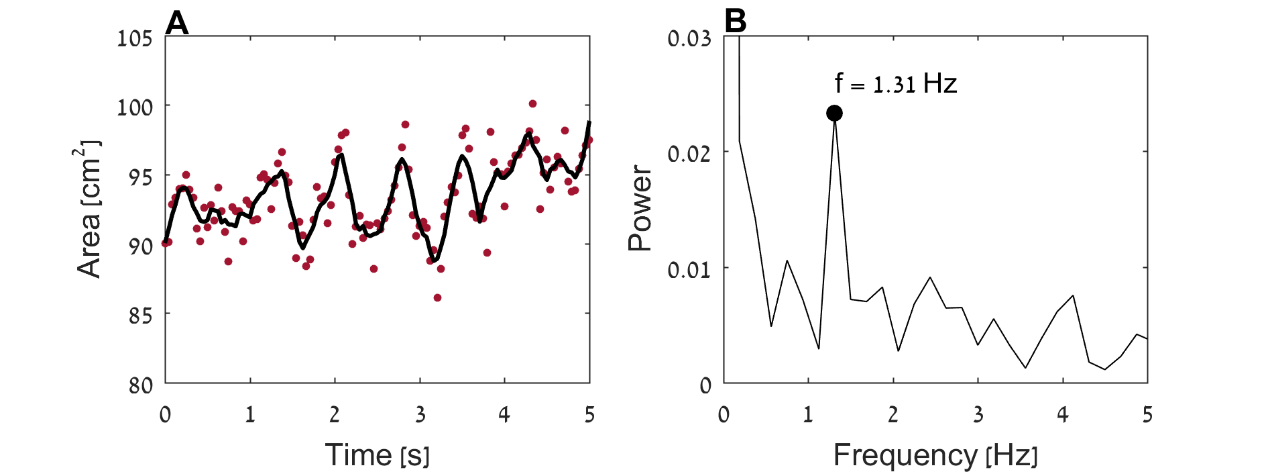


**Fig. S3. An example of the procedure used for acquiring the pulsation frequency.** (A) Projected surface area of the jellyfish image with time. Raw data (Red circle), moving average (black line; span of 5). (B) Power-frequency plot acquired via Fast Fourier Transform (FFT). The prominent frequency is marked with a black dot.

[
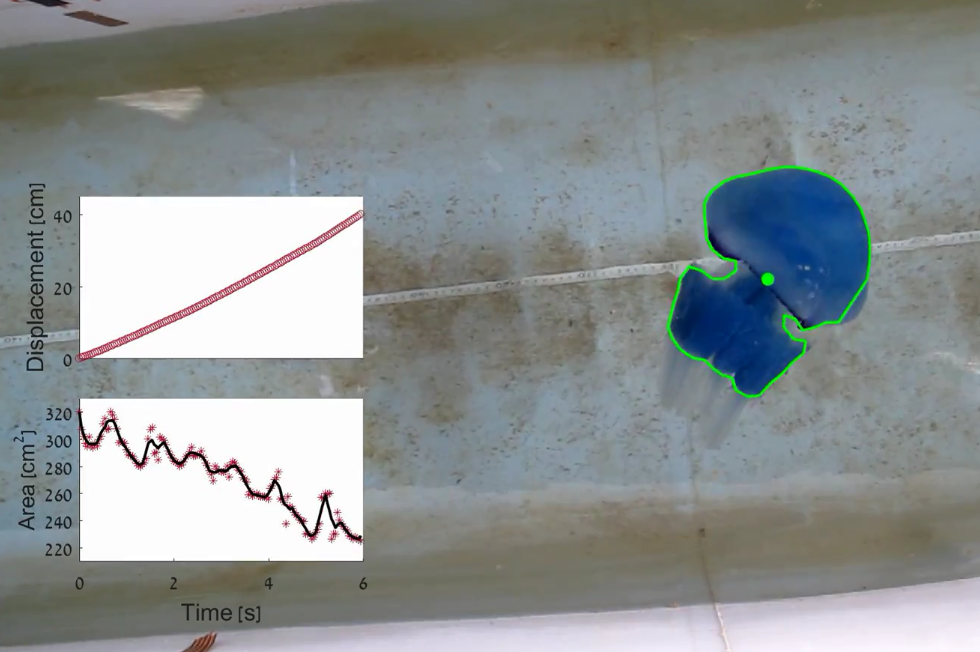
](https://www.youtube.com/watch?v=IT671fcX6to)

[**Movie S1**](https://www.youtube.com/watch?v=IT671fcX6to) **(**[**https://www.youtube.com/watch?v=IT671fcX6to**](https://www.youtube.com/watch?v=IT671fcX6to)**). Automatic outline and location tracking result.** The video displays the automatically acquired outline of the jellyfish (green line) and location (green dot) at real time and slowed down five times. The inset plots show the projected surface area (red – raw data, black line – moving average with a span of 5) and cumulative displacement over time.

*Weighted average and standard deviation*

Swimming speed ($U_{k} [L T^{-1}]$) and pulsation frequency ($f_{k}[T^{-1}]$) for the different flume flow conditions and jellyfish size (*R. pulmo*) were weighted averaged by the segments' time duration ($t_{k}[T]$). The formulations of the average swimming speed ($\bar{U}_{jelly}$), pulsation frequency ($\bar{f}_{jelly}$) and the respective standard deviations ($SD_{U}$ and ${SD}_{f}$ ) were calculated as follows:

| $\bar{U}_{jelly}=\frac{\sum_{m=1}^{M} t_{k}U_{k}}{\sum_{k=1}^{M} t_{k}}$ | (S2) |
| --- | --- |
| $\bar{f}_{jelly}=\frac{\sum_{m=1}^{M} t_{k}f_{k}}{\sum_{k=1}^{M} t_{k}}$ | (S3) |
| $SD_{U}=\sqrt{\frac{M\sum_{k=1}^{M} t_{k}\left( U_{k}-\bar{U}_{jelly} \right)^{2}}{\left( M-1 \right)\sum_{k=1}^{M} t_{k}}}$ | (S4) |
| $SD_{f}=\sqrt{\frac{M\sum_{k=1}^{M} t_{k}\left( f_{k}-\bar{f}_{jelly} \right)^{2}}{\left( M-1 \right)\sum_{k=1}^{M} t_{k}}}$ | (S5) |

where $M$ is the number of swimming segments used to calculate the average (as appears in Table S1), $t_{k}, f_{k} and U_{k}$ are the time duration of the segment, segment frequency and segment velocity of the $k$^th^ swimming segment, respectively.

**Table S2. ANOVA tables for the four separate multiple regression tests conducted.** The effect of jellyfish diameter and flume water velocity (independent variables) on swimming speed and the effect of jellyfish diameter and flume water velocity on the pulsation frequency were tested for each of the two species. Asterisks indicate levels of statistical significance: (*) $P<0.05$ (**) $P<0.01$ (***) $P<0.001$. SE is standard error of the estimate.

|  | **Estimate** | **SE** | **P value** |
| --- | --- | --- | --- |
| *Rhizostoma pulmo* swimming speed | | | |
| **Intercept** | 6.52 | 0.72 | 6.61e-16*** |
| **Diameter** | 0.14 | 0.05 | 0.0068** |
| **Water velocity** | -0.18 | 0.038 | 3.1e-06*** |
| $R^{2}=0.15, F_{2,156}=15.39$ | | | |
| *Rhizostoma pulmo* pulsation frequency | | | |
| **Intercept** | 1.75 | 0.06 | <2e-16*** |
| **Diameter** | -0.036 | 0.0042 | 5.06e-15*** |
| **Water velocity** | 0.0054 | 0.0032 | 0.09 |
| $R^{2}=0.34, F_{2,150}=39.55$ | | | |
| *Rhopilema nomadica* swimming speed | | | |
| **Intercept** | 4.39 | 1.71 | 0.013* |
| **Diameter** | 0.19 | 0.15 | 0.21 |
| **Water velocity** | -0.34 | 0.067 | 4.74e-06*** |
| $R^{2}=0.34, F_{2,51}=14.61$ | | | |
| *Rhopilema nomadica* pulsation frequency | | | |
| **Intercept** | 1.55 | 0.12 | <2e-06*** |
| **Diameter** | -0.027 | 0.01 | 0.013* |
| **Water velocity** | 0.0057 | 0.0047 | 0.23 |
| $R^{2}=0.11, F_{2,51}=4.35$ | | | |

**References**

1. Joblove, George H and Greenberg, D. Color spaces for computer graphics. *ACM siggraph Comput. Graph.* **12,** 20–25 (1978).

2. Haralick, R. M., Sternberg, S. R. & Xinhua Z. Image analysis using mathematical morphology. *IEEE transactions on pattern analysis and machine intelligence.* **4,** 532–550 (1987).

3. Huang, T. S., Yang, G. J. & Tang, G. Y. A Fast Two-Dimensional Median Filtering Algorithm. *IEEE Trans. Acoust.* **27,** 13–18 (1979).

4. Maini, R. & Himanshu A. Study and Comparison of Various Image Edge Detection Techniques. *International journal of image processing****.* 3** (1), (2008)

5. Gonzalez R. C., Woods R. E. & Eddine S. *Digital Image Processing Using MATLAB® (2^nd^ ed.).* (Gatesmark, 2009).
